# Bilateral orbital involvement of IgG4-related disease detected on ^18^F-Fluoro-2-deoxy-D-glucose positron emission tomography/computed tomography: A Clinical Case Report

**DOI:** 10.1097/MD.0000000000018138

**Published:** 2019-11-22

**Authors:** Ping Dong, Li Wang, Lin Li

**Affiliations:** a Department of Nuclear Medicine; b Department of Pancreatic Surgery, West China Hospital, Sichuan University, Chengdu, PR China.

**Keywords:** ^18^F-FDG PET/CT, bilateral orbits, IgG4-related disease, prostate

## Abstract

**Rationale:**

IgG4-related disease (IgG4-RD) is a systemic immune-mediated fibro-inflammatory condition, which could involve multiple structures, including the pancreas, salivary glands, and lymph nodes. However, cases of IgG4-RD involving the bilateral orbits, salivary glands, submandibular glands, lymph nodes, and prostate are rare.

**Patient concerns:**

A 51-year-old man complaining of bilateral exophthalmos, reduced vision, and weight loss of 15 kg over 2 years presented to our department for evaluation.

**Diagnoses:**

Based on the elevated serum IgG4 level, postoperative pathology, and the features of ^18^F-fluoro-2-deoxy-D-glucose (^18^F-FDG) positron emission tomography/computed tomography (PET/CT), which revealed diffuse increased FDG uptake in many structures, he was diagnosed with IgG4-related disease involving the bilateral orbits, salivary glands, submandibular glands, lymph nodes, and prostate.

**Interventions:**

Because of the significant bilateral proptosis and exposure keratoconjunctivitis in the right eye, bilateral soft-tissue masses located in the orbits were resected, and the patient was started on oral methylprednisolone with gradual tapering.

**Outcomes:**

The patient's symptoms gradually relieved after the operation and glucocorticoid therapy. Four months later, cranial axial CT revealed remarkable narrowing of soft-tissue masses in the bilateral orbits, and his serum IgG4 level reduced sharply.

**Lessons:**

IgG4-RD should be considered in cases of diffuse FDG uptake in the bilateral orbits, salivary glands, submandibular glands, lymph nodes, and prostate on PET/CT.

## 1 Introduction

IgG4-related disease (IgG4-RD) is a systemic immune-mediated fibro-inflammatory condition, characterized by tumor-like swelling, with variable degrees of “storiform” fibrosis, and lymphoplasmacytic infiltration enriched with IgG4-positive plasma cells.^[1–4]^ It has been found in multiple locations, including the pancreas, biliary tract, lacrimal and salivary glands, lymph nodes, retroperitoneum, kidney, thyroid, and mediastinum.^[3–8]^ The pancreas, lymph nodes, and salivary glands are most commonly involved structures in IgG4-RD.^[9]^ Although the presentation of IgG4-RD on ^18^F-fluoro-2-deoxy-D-glucose positron emission tomography/computed tomography (^18^F-FDG PET/CT) has been reported, cases of IgG4-RD involving the bilateral orbits, salivary glands, submandibular glands, lymph nodes, and prostate with intense diffuse FDG uptake are rare.^[5–9]^

## 2 Case presentation

**Figure 1:**
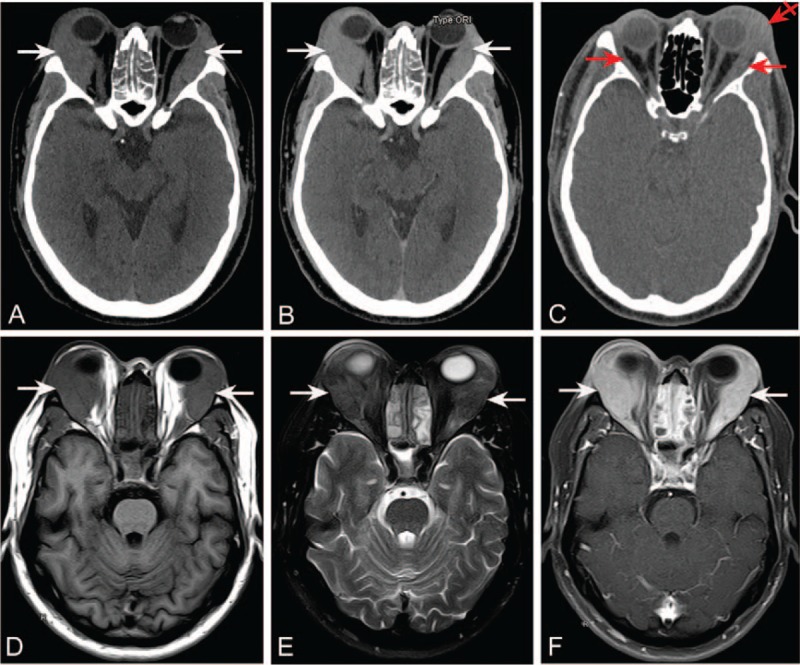
Cranial axial CT (A: non-enhanced image; B: enhanced image) and MRI (D: T1WI-FLAIR; E: T2WI; F: contrast-enhanced T1WI) demonstrate significant homogeneously enhancing soft-tissue masses (white arrows) in the bilateral orbits and crowding of the optic nerves, especially on the right side. Cranial axial CT four months later (C: non-enhanced image) reveals remarkable narrowing of the bilateral soft-tissue masses (red arrows) and slight enlargement of the left lacrimal gland (red crossed arrow). CT = computed tomography; MRI = magnetic resonance imaging; T1WI = T1-weighted imaging; FLAIR = fluid-attenuated inversion recovery; T2WI = T2-weighted imaging.

A 51-year-old man presented with bilateral exophthalmos, reduced vision, and weight loss of 15 kg over 2 years. He had a history of type 2 diabetes, which was well-controlled with metformin. Physical examination revealed decreased visual acuity of 0.5 in the right eye and 0.2 in the left eye, elevated intraocular pressure of 22 mmHg in the right eye and 23 mmHg in the left eye, bilateral exophthalmos, ocular motility disturbance, and exposure keratoconjunctivitis in the right eye. Contrast-enhanced cranial axial CT (Fig. 1 A and B) and magnetic resonance imaging (MRI) (Fig. 1 D–F) demonstrated significant homogeneously enhancing soft-tissue masses (white arrows) in the bilateral orbits and crowding of the optic nerves, especially on the right side. He was tentatively diagnosed with orbital tumor based on the CT and MRI findings. The serum tumor marker test showed slightly elevated carbohydrate antigen 72–4 and neuron-specific enolase levels at 7.18 U/mL (reference range, < 6.5 U/mL) and 16.21 ng/mL (reference range, < 15 ng/mL), respectively, but normal levels of all other tumor markers, including alpha-fetoprotein, carcinoembryonic antigen, carbohydrate antigen 15–3, carbohydrate antigen 19–9, cancer antigen 125, and total prostate-specific antigen.

**Figure 2:**
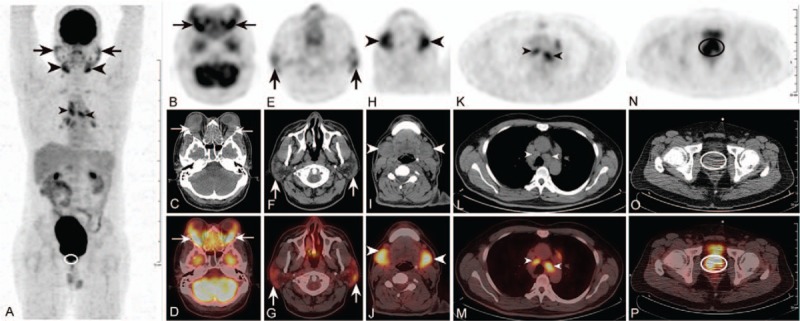
(A) The MIP image shows multiple ^18^F-FDG-avid structures. (B–P) Figures displayed from top to bottom represent axial ^18^F-FDG PET, CT, and PET/CT fusion. Whole-body ^18^F-FDG PET/CT shows increased FDG uptake in the soft-tissue masses located in the bilateral orbits (SUV_max_ of 8.21; B, C, D: thin arrows), salivary glands (SUV_max_ of 4.53; A, E, F, G: thick arrows), submandibular glands (SUV_max_ of 6.56; A, H, I, J: thick arrowheads), lymph nodes (SUV_max_ of 6.21; A, K, L, M: thin arrowheads), and prostate (SUV_max_ of 7.43; A, N, O, P: hollow ellipse), suggestive of IgG4-RD involvement in the aforementioned FDG-avid structures. MIP = maximum intensity projection; ^18^F-FDG = ^18^F-fluoro-2-deoxy-D-glucose; PET/CT = positron emission tomography/computed tomography; SUV_max_ = maximal standardized uptake value; IgG4-RD = IgG4-related disease.

Subsequently, the patient was administered ^18^F-FDG (484.3 MBq, 5 MBq/kg body weight) and imaged for 2.5 minutes per bed position, approximately 1 hour after the injection, with a Gemini 16 PET/CT scanner (Philips Healthcare, the Netherlands). Whole-body ^18^F-FDG PET/CT showed increased FDG uptake in the soft-tissue masses located in the bilateral orbits (maximal standardized uptake value [SUV_max_] of 8.21, Fig. 2B–D: thin arrows), salivary glands (SUV_max_ of 4.53, Fig. 2A, E–G: thick arrows), submandibular glands (SUV_max_ of 6.56, Fig. 2A, H–J: thick arrowheads), lymph nodes (SUV_max_ of 6.21, Fig. 2A, K–M: thin arrowheads), and prostate (SUV_max_ of 7.43, Fig. 2A, N–P: hollow ellipse). Considering the characteristics of the ^18^F-FDG uptake and the significantly elevated serum IgG4 level (8.36 g/L; reference range, 0.035–1.5 g/L), the possibility of IgG4-RD increased.

Because of the severe bilateral proptosis and exposure keratoconjunctivitis in the right eye, soft-tissue masses located in the bilateral orbits were resected. The postoperative pathology showed fibrous hyperplasia with infiltrations of many lymphocytes and plasma cells. Immunohistochemical staining revealed that the lymphocytes and plasma cells were positive for IgG4 (more than 50 cells/high-power field), IgG, CD20, CD3ε, CD23, CD138, with a Ki-67 proliferation index of about 5% to 10%, but negative for CD10 and CyclinD1. No IgH/Igκ gene rearrangement peaks were detected. Combined with the aforementioned morphological findings and immunohistochemical staining and gene rearrangement results, the diagnosis of IgG4-RD was confirmed.

The patient was started on 40 mg of oral methylprednisolone daily, which was gradually tapered. His symptoms gradually relieved after the surgery and glucocorticoid therapy. Four months later, cranial axial CT (Fig. 1C) revealed remarkable narrowing of the bilateral soft-tissue masses (red arrows) and slight enlargement of the left lacrimal gland (red crossed arrow). Further, his serum IgG4 level reduced to 0.647 g/L (reference range, 0.035–1.5 g/L).

The patient provided informed consent for publication of this case.

## 3 Discussion

IgG4-RD is a systemic immune-mediated fibro-inflammatory condition, characterized by tumor-like swelling of the involved structures, lymphoplasmacytic infiltration enriched with IgG4-positive plasma cells, and variable degrees of fibrosis, with a characteristic “storiform” pattern.^[1–4,10]^ In addition, elevated serum concentrations of IgG4 (> 135 mg/dL) are found in 60% to 70% of the patients with IgG4-RD.^[11,12]^ Although IgG4-RD occurs more commonly in middle-aged and older men, the disease extent and severity appear to be similar between men and women.^[13]^ In a series of 125 patients with biopsy-proven IgG4-RD, the number of involved organs, degree of serum IgG4 elevation, or damage from IgG4-RD did not differ between men and women.^[14]^ The pathogenesis of IgG4-RD is unclear; findings consistent with both autoimmune and allergic disorders are present.^[11,15–21]^ It has been postulated that IgG4 plays a role in tolerance to allergens and responses to certain infectious agents. However, its physiologic role is poorly understood. There is an emerging consensus that IgG4 antibodies in this disease are not pathogenic but represent a down-regulatory response to other primary processes instead.^[22]^

IgG4-RD can involve one or multiple structures (60%–90%), including the pancreas (autoimmune pancreatitis [AIP]), biliary tract (sclerosing cholangitis), lacrimal and salivary glands (Mickulicz's disease and sclerosing sialadenitis), lymph nodes (lymphadenopathy), retroperitoneum (retroperitoneal fibrosis), kidney (interstitial nephritis), thyroid (Riedel thyroiditis), and mediastinum (fibrosing mediastinitis).^[3–8,23,24]^ The pancreas, lymph nodes, and salivary glands are the most commonly involved structures in IgG4-RD.^[9]^ In addition, the orbit is also commonly involved (17%–23%) in IgG4-RD, called IgG4-related ophthalmic disease, including IgG4-related dacryoadenitis and IgG4-related orbital myositis.^[2,3,9,25]^ The typical ocular manifestation is proptosis or swelling of the ocular region, most commonly caused by dacryoadenitis (lacrimal gland enlargement). Orbital myositis (involvement of the extraocular muscles), orbital pseudotumors (without lacrimal gland invasion), or a combination of these disorders can also result in exophthalmos.^[25]^ The less common ocular manifestations of IgG4-RD are scleritis, obstruction of the nasolacrimal duct, and compression of the infraorbital and trigeminal nerves.^[26–29]^ In our patient, the primary ocular manifestations were bilateral proptosis and reduced vision. In addition, the salivary glands, submandibular glands, lymph nodes, and prostate were involved.

The diagnosis of IgG4-RD is based on the characteristic histopathologic findings on biopsy and immunohistochemical staining results, including lymphoplasmacytic tissue infiltration of mainly IgG4-positive plasma cells and lymphocytes, accompanied by fibrosis, with storiform features, and often accompanied by obliterative phlebitis and modest tissue eosinophilia.^[10,30]^ Although elevated serum IgG4 levels are not diagnostic, the serum IgG4 level should be measured because isolated elevated levels are a significant diagnostic aid.^[31,32]^

Although the diagnosis of IgG4-RD requires the presence of characteristic findings in the biopsy specimen of the affected tissues, additional organ involvement can be identified through a careful history, physical examination, routine laboratory testing, and selected imaging.^[30]^ Generally, CT of the chest, abdomen, and pelvis are performed for patients diagnosed with IgG4-RD because of the high frequency of subclinical disease.^[30]^ If available, ^18^F-FDG PET/CT can also be highly effective in determining the disease extent and should be considered at baseline.^[9,30,33]^ A prospective cohort study evaluating the value of ^18^F-FDG PET/CT in characterizing IgG4-RD revealed that ^18^F-FDG PET/CT can reveal more organ involvement compared to conventional evaluations, including physical examination, ultrasonography, and CT.^[9]^ Moreover, comprehensive understanding of all involvement can aid the biopsy-site selection.^[9]^ In addition, ^18^F-FDG PET/CT can monitor the therapeutic response after 2–4 weeks of steroid-based therapy.^[9]^ The specific characteristics and pattern of IgG4-RD on ^18^F-FDG PET/CT include diffusely elevated FDG uptake in multiple structures, including the pancreas, salivary glands, and retroperitoneal region.^[9,30]^ Most patients with IgG4-RD respond to glucocorticoids within several weeks, typically with symptomatic improvement, reduction in the size of masses or organ enlargement, improvement in organ function, and often, reduction in serum IgG4 levels.^[33,34]^ The natural history of IgG4-RD has not been well-defined. Some cases improve temporarily without treatment, but most cases progress at variable rates.^[11,34]^ In 2010, Vege et al reported that 47% of the patients with type 1 AIP (the first disease recognized to be associated with IgG4-RD) experienced a relapse and that diffuse pancreatic swelling and proximal biliary involvement can predict recurrence.^[24]^

In conclusion, we presented rare ^18^F-FDG PET/CT images with intense diffuse FDG uptake in the bilateral orbits, salivary glands, submandibular glands, lymph nodes, and prostate. This report suggests that IgG4-RD should be considered in cases of diffuse FDG uptake in the bilateral orbits, salivary glands, submandibular glands, lymph nodes, and prostate on PET/CT. In addition, ^18^F-FDG PET/CT is a useful diagnostic modality for patients with IgG4-RD, which can evaluate organ involvement, aid in biopsy-site selection, and monitor the therapeutic response.

## Author contributions

**Data curation:** Ping Dong.

**Resources:** Ping Dong, Li Wang.

**Supervision:** Lin Li.

**Writing–original draft:** Ping Dong, Li Wang.

**Writing–review & editing:** Li Wang, Lin Li.
